# Synergistic Antiviral Effects of Metal Oxides and Carbon Nanotubes

**DOI:** 10.3390/ijms231911957

**Published:** 2022-10-08

**Authors:** Indrani Gupta, Samar Azizighannad, Edgardo T. Farinas, Somenath Mitra

**Affiliations:** Department of Chemistry and Environmental Science, New Jersey Institute of Technology, Newark, NJ 07102, USA

**Keywords:** MS2 bacteriophage, nickel (II) hydroxide, iron (III) oxide, manganese (II) oxide, carbon nanotubes (CNTs), metal oxide-CNTs, antiviral activity

## Abstract

In this research, the synergistic antiviral effects of carbon nanotubes (CNTs) and metal oxides (MO) in the form of novel hybrid structures (MO-CNTs) are presented. Raw CNTs, Ni(OH)_2_, Fe_2_O_3_ and MnO_2_, as well as Ni(OH)_2_-CNT, Fe_2_O_3_-CNT and MnO_2_-CNT were explored in this study against *Escherichia. coli* MS2 bacteriophage, which was used as a virus surrogate. The nano particles were synthesized and characterized using field emission scanning electron microscopy (FESEM), energy-dispersive X-ray spectroscopy (EDS), transmission electron microscopy (TEM), particle size analysis, Fourier-transform infrared spectroscopy (FTIR) and X-ray diffraction (XRD). Kinetic parameters such as the LD_50_ (lethal dose to kill 50% of the population), T_50_ and T_80_ (time taken to kill 50% and 80% of the population), SGR (specific growth rate) and IRD (initial rate of deactivation of the population) were also studied to examine the antiviral efficacy of these nanomaterials. Among all the nanomaterials, Ni(OH)_2_-CNT was the most effective antiviral agent followed by Fe_2_O_3_-CNT, MnO_2_-CNT, raw CNTs, Ni(OH)_2_, Fe_2_O_3_ and MnO_2_. When comparing the metal oxide-CNTs to the raw CNTs, the average enhancement was 20.2%. The average antiviral activity enhancement of the MO-CNTs were between 50 and 54% higher than the MO itself. When compared to the raw CNTs, the average enhancement over all the MO-CNTs was 20.2%. The kinetic studies showed that the LD_50_ of Ni(OH)_2_-CNT was the lowest (16µg/mL), which implies that it was the most toxic of all the compounds studied. The LD_50_ of Ni(OH)_2_, Fe_2_O_3_ and MnO_2_ were 17.3×, 14.5× and 10.8× times greater than their corresponding hybrids with the CNTs. The synergistic mechanism involved the entrapment of phage viruses by the nano structured CNTs leading to structural damage along with toxicity to phage from the release of MO ions. The metal oxide-CNT nano hybrids developed in this project are promising candidates in applications such as antiviral coatings, nanocomposites, adsorbents and as components of personal protection gears.

## 1. Introduction

Pathogen-based infections are a major cause of different infections across the world which pose a threat to human health [1]. The conventional method of treating pathogens is through the use of radiation or chemical disinfectants [2]. Although chemical disinfectants are relatively inexpensive and easy to use, they generate toxic byproducts, have shorter lifetimes, may be corrosive and a high dosage may be required to obtain 100% efficiency [3,4]. Moreover, these cannot be embedded in polymeric and other matrices. The recent spread of COVID-19 has demonstrated the need for antiviral nanomaterials which can be embedded as components of personal protective equipment, adsorbents and be a medium for air and water purification [5]. Metal nanoparticles (M-NPs) have gained importance as antimicrobial agents due to their unique physicochemical properties which allow them to interact with microorganisms [6,7]. These properties include a smaller dimension (usually between 1 and 100 nm) and a large surface area for an enhanced interaction [8,9]. The antiviral activity of the nano particles consists of different types of interactions such as attaching to the viral surface to inactivate the functionality of DNA/RNA, penetration into host cells to destroy its structure and generating reactive oxygen species [2,10].

Besides M-NPs, metal complexes have proven to be efficient as well [7]. For example, metal-oxide NPs (MO-NPs) have received much attention due to their higher stability, easy engineering to obtain the required shape/size/porosity, easy functionalization and integration into hydrophobic/hydrophilic systems for biomedical applications [2,11,12]. Among all the MO-NPs, transition metals make the most effective antimicrobial agents [13] and the MOs of silver, zinc, aluminum and titanium have been successfully used [14]. Nickel, manganese and iron MO-NPs have shown antiviral activity against H1N1 influenza A virus, hepatitis C virus, human immunodeficiency virus and vesicular stomatitis virus [15,16,17,18,19]. In general, their antimicrobial mechanism predominantly depends on (1) size; (2) stability; and (3) concentration in the solution. Since MO-NPs are usually smaller than most microbes, they have the unique ability to permeate through the outer membrane of microbes [20].

Besides M-NPs and MO-NPs, nanocarbons [21,22,23] such as carbon nanotubes (CNTs) have also been used as antimicrobial agents [20,22]. CNTs are allotropes of carbon with great hydrophobicity and have been used in various other fields such as solvent enrichment [24,25,26,27,28], fouling reduction [29,30] and hydrocarbon dewatering [31,32]. Extensive research has been performed on CNTs to explore their antimicrobial properties [33,34]. The factors that play a role in its antimicrobial activity include its size and surface area [35]. The mechanism of action for CNTs have been attributed to a number of reasons. These include microbial adhesion (wrapping around) to the nanocarbons leading to the interruption of a transmembrane electron transfer and causing membrane disruption, physical puncture into microbes resulting in DNA/RNA damage and protein dysfunction and (3) the generation of reactive oxygen species (ROS) [36,37].

As of now, there are very few reports related to the antiviral properties of hybrid nanomaterials. Recently, our group has reported the antibacterial properties of CNTs using *E. coli* and *G. stearothermophilus* strains, and the antiviral properties of different functionalized CNTs using bacteriophages as a surrogate [38,39,40]. It is believed that the strong physical and chemical interactions between CNTs and MO can complement each other and lead to enhanced antiviral activity. The objective of this research was to investigate if there are synergistic effects between CNTs and MOs to enhance antiviral efficiency.

## 2. Results and Discussion

### 2.1. Sample Characterization

**Figure 1:**
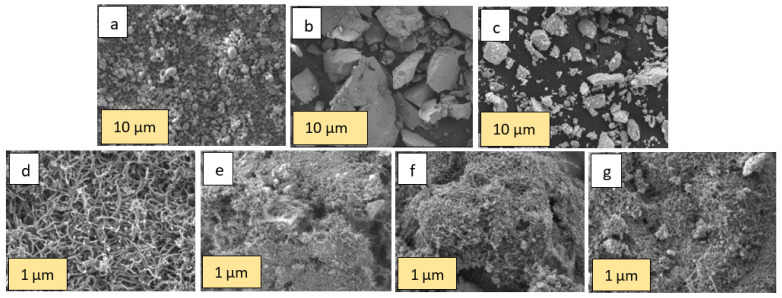
SEM image of (**a**) Ni(OH)_2_; (**b**) Fe_2_O_3_; (**c**) MnO_2_; (**d**) CNTs; (**e**) Ni(OH)_2_-CNT; (**f**) Fe_2_O_3_-CNT; and (**g**) MnO_2_-CNT.

The prepared nanomaterials were characterized using field emission scanning electron microscopy (FESEM) (JEOL; model JSM-7900F), transmission electron microscopy (TEM) (JEOL; model JEM-F200), energy dispersive X-ray spectroscopy (EDS) (JEOL; model JSM-7900F), particle size determination through a dynamic light scattering (DLS) technique (Malvern Nano ZS DLS), Fourier-transform infrared spectroscopy (FTIR) (Shimadzu, IRAffinity-1) and X-ray diffraction (XRD) (Pan-analytical Empyrean XRD instrument with Cu Kα radiation source under scanning conditions of 10–100°). Figure 1a–g show the FESEM images of the MOs, CNT and MO-CNT hybrids, namely Ni(OH)_2_, Fe_2_O_3_, MnO_2_, CNTs, Ni(OH)_2_-CNT, Fe_2_O_3_-CNT and MnO_2_-CNT, respectively. Figure 1a–c show the Ni(OH)_2_, Fe_2_O_3_ and MnO_2_ particles, respectively. Under the FESEM, these particles appeared as agglomerated structures and its exact morphology could not be defined. Additionally, the particle size of the nanomaterials was highly variable, with Ni(OH)_2_ exhibiting the lowest particle size distribution. Figure 1d–g show the CNTs and the MO-CNTs. There was a clear distinction between the threaded structures of the raw CNTs in Figure 1d and the small particles (which appear as white particles) of the MOs embedded in the tubular structures of the raw CNTs in Figure 1e–g.

**Figure 2:**
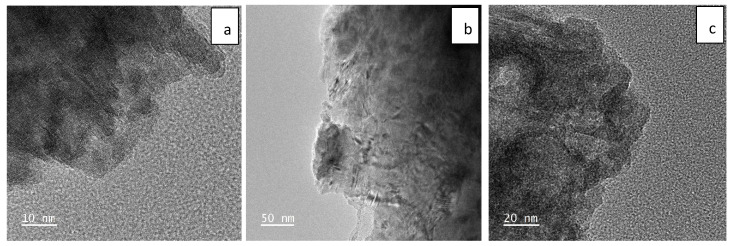

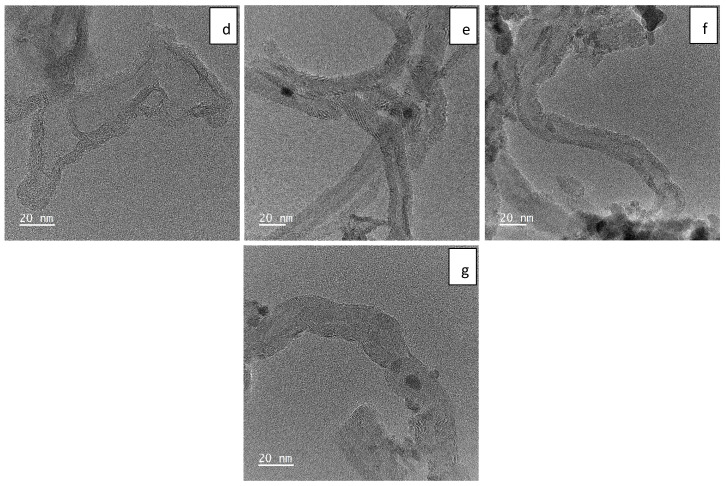
TEM image of (**a**) Ni(OH)_2_; (**b**) Fe_2_O_3_; (**c**) MnO_2_; (**d**) CNTs; (**e**) Ni(OH)_2_-CNT; (**f**) Fe_2_O_3_-CNT; and (**g**) MnO_2_-CNT.

Besides FESEM, TEM was also performed to obtain a more detailed imaging of the nanomaterials at near-atomic resolution. Figure 2a–g show the TEM images of Ni(OH)_2,_ Fe_2_O_3,_ MnO_2,_ CNTs, Ni(OH)_2_-CNT, Fe_2_O_3_-CNT and MnO_2_-CNT, respectively. Figure 2a shows the crystal structure of the layered Ni(OH)_2_ sheet-like NPs. It appears that these layers were stacked on top of one another and had sharp edges. The thickness between the layers was measured to be less than 1 nm. Figure 2b shows the Fe_2_O_3_ which had a 50:50 crystalline/amorphous structure. The sharp black strokes on the transparent Fe_2_O_3_ indicated a wrinkled appearance. Moreover, the TEM results of MnO_2_ show that these were aggregates that formed a block with a porous structure. The MnO_2_ particles in Figure 2c were completely amorphous. The raw CNTs in Figure 2d show their multiwalled structure. These CNTs had an internal and external diameter of 0.5–1 nm and 12–15 nm, respectively. The Mos can be seen as black particles distributed on the CNT structures in Figure 2e–g.

Figure S1 (in the Supplementary Information) shows the EDS spectra of the MOs and MO-CNTs, and Table 1 lists the elemental composition of the MOs and MO-CNTs used in this study. However, this composition is not uniform as it is a site-specific measurement and the average values out of three measurements have been reported. This test was mostly carried out to confirm the presence of the elements in the MOs and the MO-CNTs as well.

**Figure 3:**
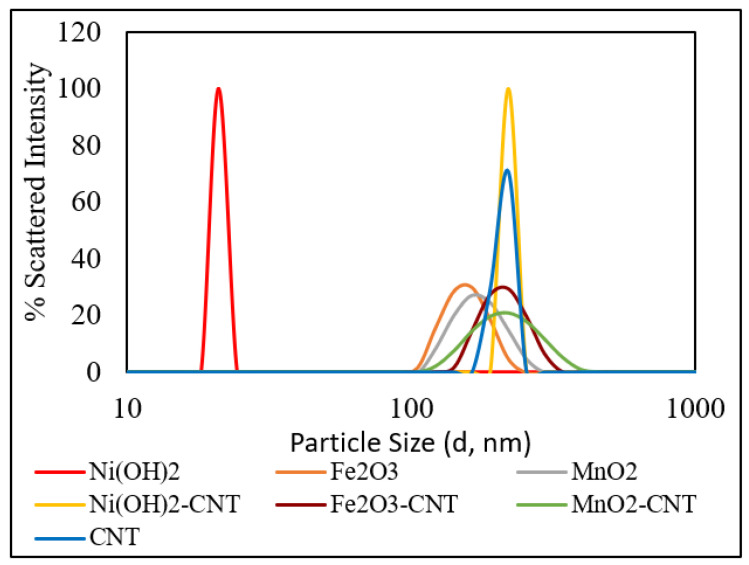
Particle size distribution of the MOs, CNTs and MO-CNT hybrids.

Particle size distribution based on the DLS measurements is presented in Figure 3. The backscattering angle was fixed at 90°. The nanomaterials were diluted in Milli-Q water and ultrasonicated for 1 h for uniform dispersion. As can be seen from Figure 3, Ni(OH)_2_ had an average particle size of 21 nm, followed by Fe_2_O_3_ NPs at 142 nm, MnO_2_ NPs at 164 nm, raw CNTs and MO-CNTs at 220 nm. As the MOs were smaller in size than the CNTs, the particle sizes of the MO-CNTs did not change after the incorporation of the MOs into the CNTs. The polydispersity index (PDI) of all the nanomaterials were between 0.2 and 0.4. Among all the nanomaterials, the PDI was the lowest for Ni(OH)_2_ and Ni(OH)_2_-CNT.

**Figure 4:**
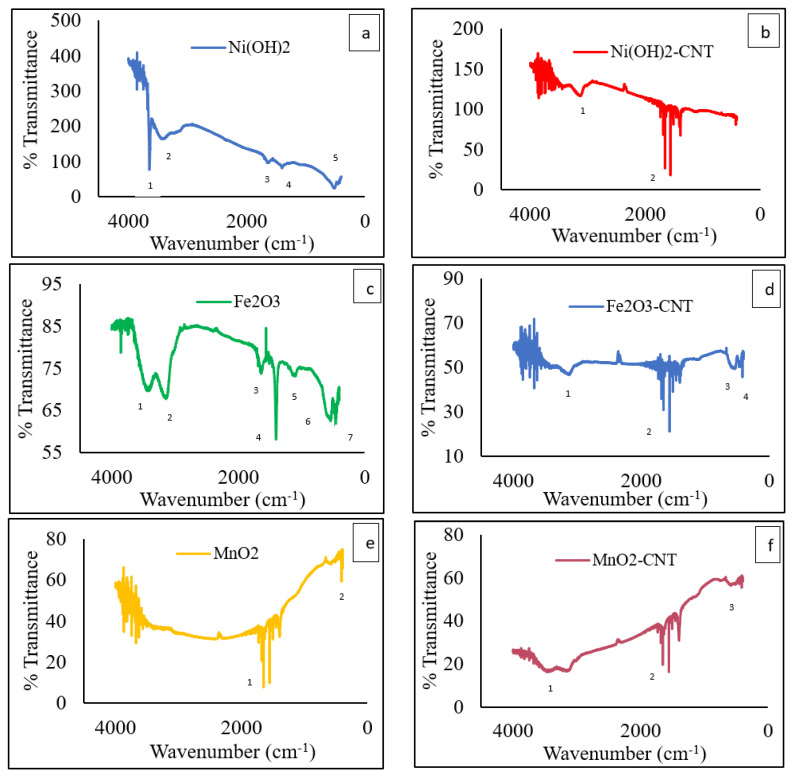
FTIR Analysis of (**a**) Ni(OH)_2_; (**b**) Ni(OH)_2__CNT; (**c**) Fe_2_O_3_; (**d**) Fe_2_O_3__CNT; (**e**) MnO_2_; and (**f**) MnO2_CNT.

The FTIR analysis of the MOs and MO-CNT hybrids is shown in Figure 4. Figure 4a shows the vibrational peaks of Ni(OH)_2_ at 3643 (labelled as 1) and 3392 cm^−1^ (2) due to the stretching of O–H bonds. Two bending vibrations were observed at 1642 (3) and 1398 cm^−1^ (4) due to adsorbed water molecules and another bending vibration was observed at 509 cm^−1^ (5), which was attributed to Ni–O stretching and Ni–O–H stretching [41]. In Figure 4b, there was a single stretching of the O–H bond at 3120 cm^−1^ (1) and a few bands between 1384 and 1652 cm^−1^ (2), which corresponded to a carbonyl (C=O) stretch. This was probably due to the presence of a carboxylic acid functional group from the added CNTs. These bands were observed in all the MO-CNTs in Figure 4b,d,f. From Figure 4c, the stretching bands at 3406 (1) and 3147 (2) cm^−1^ belonged to the O–H functional groups. The sharp peak at 1400 cm^−1^ (4) and a low intensity peak observed at 1633 cm^−1^ (3) were due to the H–O–H adsorbed water frequency, as reported before [42]. The peak at 1093 cm^−1^ (5) was due to OH stretching vibration [42]. Additionally, there was a broad and one sharp band in the range of 536 (6) and 457 (7) cm^−1^, respectively, which were attributed to the Fe–O bonds [43,44] and these bands were also observed in Figure 4d as (3) and (4), respectively. The O–H band stretching significantly decreased in Figure 4d (1 & 2) (Fe_2_O_3_-CNT) and this could be attributed to the conversion of the O–H functional group to oxides which led to the formation of C=O bands between 1500 and 1600 cm^−1^. The FTIR spectrum of MnO_2_ (Figure 4e) exhibited the C=O stretching vibrations at 1558 and 1652 cm^−1^ (1). A sharp bend at 418 cm^−1^ (2) represented the Mn-O stretching vibration [45] which was also observed in Figure 4f as (3). MnO_2_-CNT also exhibited a C=O stretching vibration at 1558 and 1652 cm^−1^ as (2). It has an additional broad stretching vibration at 3113–3415 cm^−1^ (1) which was attributed to the O–H functional group.

**Figure 5:**
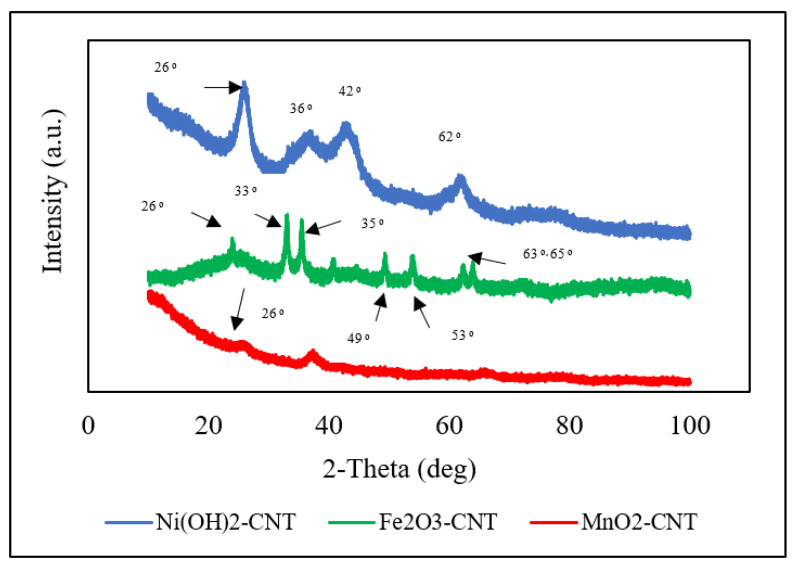
XRD spectra of MO-CNT hybrids.

The XRD analysis of the MO-CNTs are presented in Figure 5. The CNTs are known to have a characteristic graphite peak at 26° [46], which corresponds to C (002) and can be seen in all the MO-CNTs plots, indicating the successful incorporation of MOs into the CNTs with negligible damage to the CNT structures [47]. The (002) peak also confirmed the multiwalled nature of the CNTs. Among the three MO-CNTs, Ni(OH)_2_-CNT had the maximum number of high intensity peaks with a moderate sharpness located at 36°, 42° and 62° which indicated that it was more crystalline than the other two. These peaks corresponded to the (002), (110) and (300) diffraction planes, respectively [48]. These peaks confirmed the successful preparation of Ni(OH)_2_-CNT and the absence of any impurity. The diffractogram of MnO_2_-CNT hardly showed any peak. A low intensity and broad peak were observed at 38° which can be indexed to a birnessite-type MnO_2_ and was be similar to what has been published in the literature [49]. Additionally, it was quite evident that it was mainly amorphous. The distinct peaks of Fe_2_O_3_ were found at 2𝜃 of 33°, 35°, 40°, 49°, 53°, 63° and 65°, indicating a crystalline structure [47]. The diffraction patterns at 33°, 35° and 63° correspond to (220), (311) and (440) diffraction planes according to the literature (JCPDS 04-0755) [50], but lower intensities indicated the successful incorporation of the Fe_2_O_3_ into the CNTs while retaining its structure [51].

### 2.2. Antiviral Effects of MOs and MO-CNT Hybrids

**Figure 6:**
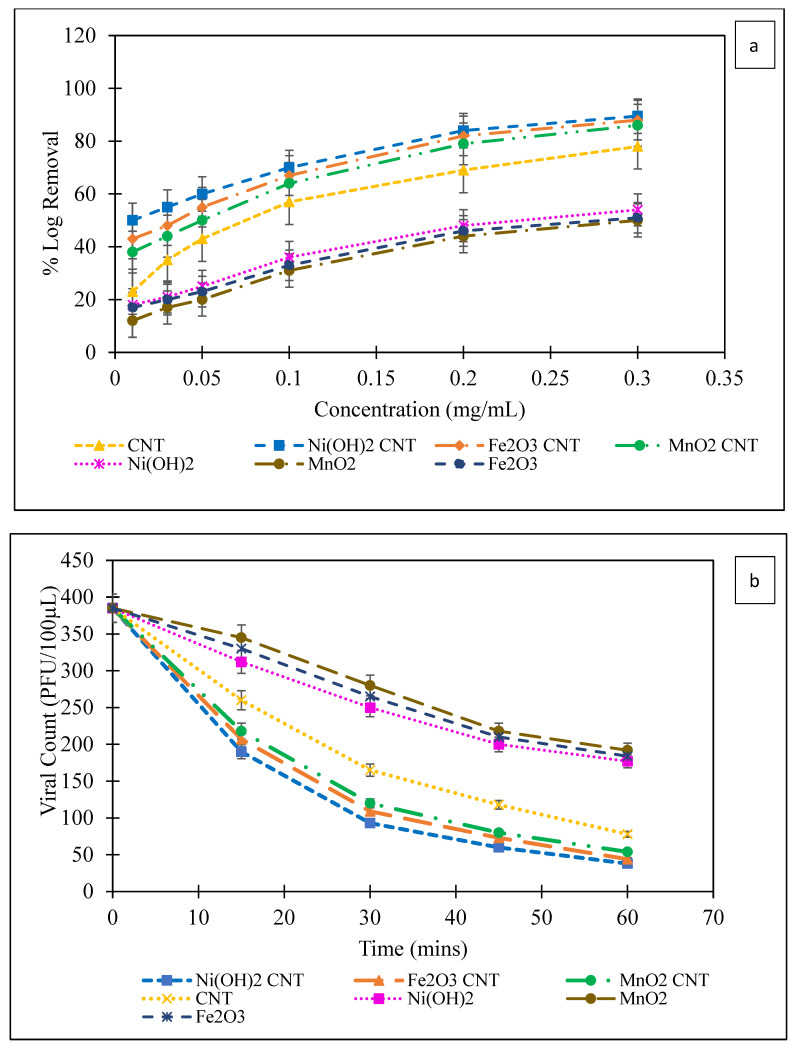
(**a**) Percentage log removal of MS2 phages at different concentrations of MO and MO-CNT; (**b**) MS2 phage reduction kinetics at 0.3mg/mL.

The experiments were performed with raw CNTs, MOs and MO-CNTs. The efficiency of the phage virus deactivation of MOs and MO-CNTs is presented in Figure 6a,b. The deactivation of the phage viruses was expressed as:

(1) $$Log\;Removal=log_{10}\left(\frac{A}{B}\right)\;$$

where *A* and *B* refer to the number of phage viruses before and after the experiment. The percentage log removal of the phage viruses against the nanomaterials is shown in Figure 6a. In general, it was observed that with an increase in concentration, the antiviral efficiency increased as well, and the slope of the curve started to smooth out beyond 0.1 mg/mL. The raw CNTs functioned better than the Mos and were 1.5 times more effective than the Mos at the highest concentration studied (0.3 mg/mL). Among the Mos, the efficiency of Ni(OH)_2_ was slightly higher than Fe_2_O_3_ and MnO_2._ This may be due to the quicker and higher generation of hydroxyl radicals from the breakdown of Ni(OH)_2_ as compared to Fe_2_O_3_ where the intermediate compound is Fe(OH)_3_ before the generation of hydroxyl radicals. It is well known that nanometals have a higher antimicrobial property [52] than larger particles, and being immobilized on CNTs, all the metals oxides automatically were nanostructured, which led to higher antiviral effects (Figure 3).

The probable mechanism of the antiviral activity of the MOs will be discussed later in the text. The conjugation of MOs to CNTs, namely Ni(OH)_2_-CNT, Fe_2_O_3_-CNT and MnO_2_-CNT, enhanced the antiviral performance by 65%, 73% and 72%, respectively, at 0.3 mg/mL. At the highest concentration, the MOs by themselves could inactivate up to 50% of the population, whereas the MO-CNT hybrids could inactivate as much as 86–90% of the population.

The reduction kinetics in the viral population at 0.3 mg/mL is been shown in Figure 6b. The rate of viral deactivation was higher for the MO-CNTs followed by the raw CNTs and the MOs by themselves. The antiviral activity was quantified based on both the concentration of nanomaterials needed to reach a 50 percent deactivation or lethal dose 50 (LD_50_), as well as the rate of deactivation. From the reduction kinetics and the % log removal, different parameters such as the LD 50, time for 50% (T_50_) and 80% (T_80_) viral deactivation, rate of initial degradation and specific growth rate were determined and put together in Table 2.

The kinetics of virus deactivation were determined from the plots in Figure 6a,b. T_50_ and T_80_ refer to the time needed to deactivate 50% and 80% of the phage viruses, respectively. The T_50_ and T_80_ of the raw CNTs were almost 37–38% lower than the MO, implying that the time taken to kill 50% and 80% of the population was shorter by that amount than the respective MO. Among the MO, Ni(OH)_2_ was slightly faster in deactivating the phage viruses and that was attributed to the higher generation of Ni^2+^ ions in the solution. The T_50_ and T_80_ of the MO-CNT were 2.2 times lesser than the MO. The lowest T_50_ and T_80_ were recorded for Ni(OH)_2_-CNT, followed by Fe_2_O_3_-CNT and MnO_2_-CNT. The rate of deactivation could be estimated from the difference between the T_50_ and T_80_ values and was found to be fastest for the MO-CNTs, followed by the raw CNTs and then the Mos. The initial rate of deactivation (IRD) was calculated from the slope of the kinetic plot (Figure 6b) at time T_0_. The Mos had a faster initial rate of deactivation compared to the other nanomaterials, but the rate of deactivation gradually decreased, as was evident from their T_50_ and T_80_ values.

The LD_50_ was defined as the median lethal dose at which one half of the subjects in a population were killed at a particular time. The LD_50_ was calculated from Figure 6a,b and was analyzed using Probit Analysis. A lower LD_50_ implied that a low dose was sufficient to kill 50% of the population and thereby implied a stronger antiviral activity. The LD_50_ of the MOs, Ni(OH)_2_, Fe_2_O_3_ and MnO_2_ were 4.4×, 5.5× and 5.6× higher than the LD_50_ of the raw CNTs. However, the LD_50_ of Ni(OH)_2_-CNT, Fe_2_O_3_-CNT and MnO_2_-CNT reduced by 94.2%, 93% and 90.7%, respectively, making them better antiviral agents than the MOs and the CNTs. The combined effect of ion release by the MO with the antiviral properties of the CNTs played a key role in its synergistic effect on the phage viruses. 

Another important parameter was the specific growth rate (SGR) at 0.03 mg/mL of the phage virus, and was determined from the equation below:

(2) $$\ln x=µt+\ln x_{o}$$

where *x_o_* is the initial virus concentration before the experiment, *x* is the final virus concentration after the experiment, µ is the specific growth rate and *t* is the time of the experiment. It is evident from Table 2 that the presence of the nanomaterials significantly reduced the specific growth rate of the phage virus. The negative values indicate that the inactivation rate of the phage was greater than its specific growth rate. As compared to the control, the specific growth rate decreased 175 times for the MO-CNTs, 150 times for the raw CNTs and 65 times for the MOs.

The antiviral efficacy was inversely proportional to the LD_50_, T_50_, T_80_ and SGR with little dependency on the IRD. In summary, it was observed that the antiviral efficacy was the highest for Ni(OH)_2_-CNT because it had the lowest LD_50_, T_50_, T_80_ and SGR. The MOs performed more or less in a similar fashion, with a small difference in their kinetic parameters. The raw CNTs were better than the MOs. It was evident that the MO and the CNTs functioned in a synergistic manner.

### 2.3. Mechanism of Deactivation

Different inactivation mechanisms have been proposed for Mos, such as the alteration of viral attachment by displacing the viral active sites with metal ions, viral surface oxidation, the alteration of the genetic make-up through nucleic acid breakage or crosslinking or viral capsid structural deformation [53]. Usually, viruses attach to surfaces of nanoparticles via van der Waals and electrostatic interactions. Typically, van der Waals interactions are dominant mechanisms. Besides that, hydrogen bonding also plays a major role in virus adsorption to a hydroxyl-containing surface (Ni(OH)_2_). Thereafter, the metal ions may alter the structure and/or alter the function of the proteins or facilitate hydrolysis or nucleophilic displacement in the genome. An important step in considering the antiviral effect of MOs is the speciation of those MOs [54]. A change in pH can increase virus susceptibility to degradation by bringing about a structural or chemical deformation [55]. When MOs such as Ni(OH)_2,_ Fe_2_O_3,_ and MnO_2_ are added to water, hydroxyl radicals and metals in their ionic state (Ni^2+^, Fe^3+^ and Mn^2+^) are generated [56]. The hydroxyl radicals are known to inactivate viruses by completely disrupting the outer envelope of viruses through physical puncture, ultimately causing genome damage [57].

**Figure 7:**
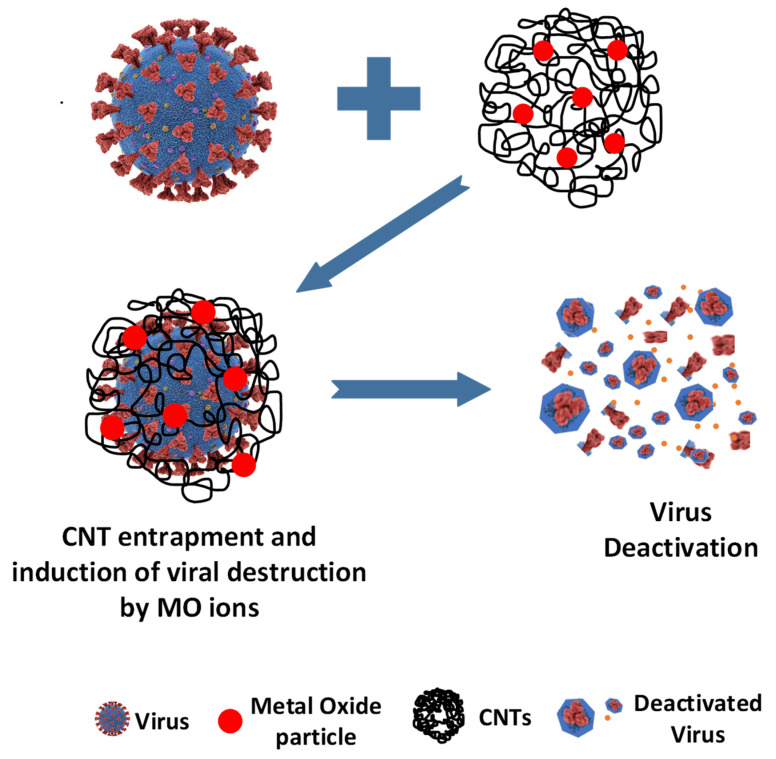
Schematic representation of proposed mechanism for phage virus inactivation.

The antiviral mechanism of CNTs have been explored in our previous study [40]. The phage viruses adhere to the CNTs via van der Waals forces. The outer surface proteins get entangled within the CNT threaded structures and the strong fibers of the CNT can cause a physical puncture, leading to the disruption of the phage virus. The proposed mechanism of deactivation is shown in Figure 7.

Virus inactivation by MO-CNTs is achieved by both physical and chemical processes. The enhanced antiviral activities of MO-CNTs are attributed to the synergistic effects of the CNTs and MO. We believe that the CNTs act as a trap for the virus particles, which may also bring about structural deformation due to entanglement. Thereafter, the MO cause a further disruption of the viral particles, leading to enhanced antiviral activity. While the CNTs bring about a strong physical disruption, the Mos provide mental ions that become a source of chemical-based deactivation.

## 3. Experimental Section

### 3.1. Chemicals and Materials

Host strain of *E. coli* C-3000 (ATCC-15597) and MS2 bacteriophage (ATCC-15597-B1) were purchased from American Type Culture Collection (ATCC), Manassas, VA, USA. PES filter (0.22 μm) was purchased from Sigma-Aldrich, St. Louis, MO, USA. Nanomaterials in this article included raw CNTs (average diameter ∼ 30 nm and a length range of 15 μm) that were obtained from Cheap Tubes Inc., Brattleboro, VT, USA; nickel hydroxide CNT (Ni(OH)_2_-CNT), ferric oxide CNT (Fe_2_O_3_-CNT) and manganese oxide CNT (MnO_2_-CNT) were prepared in the laboratory.

### 3.2. MS2 Bacteriophage Sample Preparation

In this study, *E. coli*/MS2 bacteriophage was used as the host–virus model system. MS2 bacteriophage is a non-enveloped, positive sense RNA-based bacteriophage with a size range between 23 and 28 nm that infects *E. coli* and some other bacterial strains [58]. MS2 bacteriophages are deemed as suitable prototypes because it can be easily cultured, have shared features with other eukaryotic viruses and can be purchased at a low cost [59].

The phage virus strain was obtained commercially and diluted in LB media. After dilution, 100 µL of the virus-LB suspension was added to 200 µL of a previously grown *E. coli* culture having an optical density (O.D.) between 0.2 and 0.4 and incubated at 37 °C with 150 RPM shaking overnight to maximize viral growth. The MS2-*E. coli* culture was centrifuged at 1000 RPM for 45 min to obtain a clear supernatant which was filtered through a 0.22 µm PES filter to isolate any remaining cells from the filtrate containing the viral suspension.

### 3.3. Preparation of MOs and MO-CNTs

The MOs and MO-CNTs were prepared according to procedures reported in a previous study [60]. An amount of 1.3 g of Ni(NO_3_)_2_.6HCl was dissolved in 200 mL of deionized (DI) water and stirred for 30 min. A total of 9 mL of a 1 M NaOH was then added dropwise at a rate of 1 mL/min. The mixture was allowed to react for 30 min and was washed, filtered and kept in a vacuum heating system to dry overnight to obtain Ni(OH)_2_ particles.

Pristine CNT was functionalized using a method published before [61]. It was carried out via acid treatment in a microwave reactor to generate carboxylated CNTs [61]. Ni(OH)_2_-CNT was prepared in the ratio of 4:1 where 0.3 g of the carboxylated CNT was added to 1.3 g of Ni(NO_3_)_2_.6HCl and stirred and sonicated for 1 h in total. Thereafter, 1 M NaOH was added dropwise in a similar manner and the same reaction steps were followed once again to obtain Ni(OH)_2_-CNT particles the following day.

Fe_2_O_3_ particles were prepared by mixing 180 mL of a 0.1 M Fe(NO_3_)_3_ to 28.2 mL of a 2 M NaOH (which was added dropwise at a rate of 0.8 mL/min). The sample reacted for 30 min and was washed, filtered and kept in a vacuum heating system to dry overnight. A pre-weighted CNT was sonicated in a mixture of nitric and sulfuric acid for 10 min. Fe_2_O_3_-CNT was prepared in the ratio of 3:1 by adding 0.5 g of CNT to the same amount of 0.1 M Fe(NO_3_)_3_ and was stirred and sonicated for 1 h in total. Thereafter, the same amount of 2 M NaOH was added dropwise and the reaction took place for 30 min, after which it was washed and filtered and kept in the vacuum heating system overnight for drying.

To prepare MnO_2_ particles, 50 mL of a 0.25 M KMnO_4_ was titrated against 1.15 mL of absolute ethanol solution (diluted to 10 mL) and reacted for 30 min. It was then put in the microwave oven at 120 °C for 60 min and then washed, filtered and dried to obtain MnO_2_ powder. The MnO_2_-CNT was prepared in the ratio of 8.5:1. A total of 50 mL of a 0.25 M KMnO_4_ was added to 0.128 g of CNT and stirred for 30 min. Then, 1.15 mL of absolute ethanol solution was diluted to 10 mL which was titrated against KMnO_4_-CNT solution and reacted for 30 min. Thereafter, 60% of the powdered solution was put in the microwave oven at 120 °C for 60 min and then was washed, filtered and dried to obtain MnO_2_-CNT powder.

### 3.4. Phage Deactivation

Different concentrations of 0.01 mg/mL, 0.03 mg/mL, 0.05 mg/mL, 0.1 mg/mL, 0.2 mg/mL and 0.3 mg/mL of the MOs and MO-CNTs were reacted with the phage viral suspension in 2 mL Eppendorf tubes by shaking at the growth temperature of MS2 bacteriophage (37 °C). Stirring was carried out at 150 RPM for 1 h. Samples were collected after every 15 min, centrifuged at 1000 RPM and filtered to ensure the absence of any nanomaterials in the filtrate. A total of 2.5 µL of the filtrate was then mixed with 20µL of *E. coli* culture and added to 5 mL of LB top agar solution (0.6% Agar) which was poured onto previously prepared LB-agar plates and after solidification, it was incubated at 37 °C for 24 h. The viral colonies, represented as PFU/µL, were enumerated using the density-pixel method in AlphaImager^®^ EP gel dock software (version 3.2.2.0), Cell Biosciences Inc., Santa Clara, CA, USA. In the density-pixel method, the boundaries of viral plaques are defined, its pixel number and density are assessed and its corresponding numerical data is calculated. These experiments were performed in triplicate and the average results have been presented in this study.

## 4. Conclusions

In this study, the antiviral efficacy of nickel (II) hydroxide, iron (III) oxide, manganese (II) oxide and their hybrids with CNTs was studied. The average enhancement in antiviral activity (based on phage death) of the MO-CNTs over pure Mos was over 50% at all studied concentrations. Kinetic parameters were studied to further assess the antiviral property of the nanomaterials. The LD_50_ of Ni(OH)_2_-CNT was the lowest (16µg/mL), indicating the highest antiviral activity. Based on the LD_50_ results also, it was inferred that the MOs were less toxic to the virus than their respective CNT hybrids. The difference between T_50_ and T_80_ was the highest for MnO_2_ and the lowest for Ni(OH)_2_-CNT, which represented the slowest and fastest phage deactivation among the systems studied. The synergistic mechanism of antiviral activity of the MO-CNTs involved the entrapment of the viruses by the nano-structured CNTs, followed by fast generation of metal ions, that lead to degradation and genome damage. It is expected that the antiviral MO-CNTs can find real-world applications by being embedded in personal protective equipment, adsorbents and as medium for air and water purification.

## Figures and Tables

**Table 1:** Elemental composition of the MOs and MO-CNT hybrids.

Sample	Elemental Composition				
	C (%)	O (%)	Ni (%)	Fe (%)	Mn (%)
Ni(OH)_2_	N/A	42.4	57.6	N/A	N/A
Fe_2_O_3_	N/A	11.3	N/A	88.7	N/A
MnO_2_	N/A	34.9	N/A	N/A	65.1
Ni(OH)_2_-CNT	64	20	15	N/A	N/A
Fe_2_O_3_-CNT	55	36	N/A	8	N/A
MnO_2_-CNT	54	36	N/A	N/A	9

**Table 2:** Phage virus inactivation kinetic parameters.

Nanomaterials	T_50_ (min)	T_80_ (min)	LD_50_ (µg/mL)	Specific Growth Rate (SGR) (hr^−1^)	Initial Deactivation Rate (IRD) (hr^−1^)
Ni(OH)_2_	50.6	83.4	278	−0.013	−3.52
Fe_2_O_3_	53.8	86.8	348	−0.012	−3.48
MnO_2_	56.9	90.5	357	−0.011	−3.42
CNT	31.8	54.6	63	−0.025	−5.42
Ni(OH)_2_-CNT	22.7	44.2	16	−0.038	−5.46
Fe_2_O_3_-CNT	24.3	46.0	24	−0.035	−5.44
MnO_2_-CNT	25.1	46.3	33	−0.032	−5.45

## Data Availability

Not applicable.

## Supplementary Materials

The following supporting information can be downloaded at: https://www.mdpi.com/article/10.3390/ijms231911957/s1, Figure S1. EDS spectra of (a). Ni(OH)_2_; (b). Fe_2_O_3_; (c). MnO_2_; (d). Ni(OH)_2_-CNT; (e). Fe_2_O_3_-CNT; (f). MnO_2_-CNT.
