# Supplementary material for: Synergistic Antiviral Effects of Metal Oxides and Carbon Nanotubes
Source: Int J Mol Sci. 2022 Oct 8;23(19):11957. doi: 10.3390/ijms231911957 (PMC9569974; doi:10.3390/ijms231911957)
Supplement: Supplementary file 1 [file ijms-23-11957-s001.zip › ijms-1892517-supplementary.pdf]

Supplementary Information

Synergistic Antiviral Effects of Metal Oxides and Carbon Nanotubes

Indrani Gupta, Samar Azizighannad, Edgardo T. Farinas and Somenath Mitra*

Department of Chemistry and Environmental Science,

New Jersey Institute of Technology

Newark, NJ, 07102

USA

* Corresponding Author

Somenath Mitra, 973-596-5611(t), 973-596-3586(F), somenath.mitra@njit.edu

1. Energy Dispersive X-ray Spectroscopy Studies

The EDS spectra for the MOs and the MO-CNTs have been presented in Figure S1. Figures S1a-c show the presence of Ni (57.6%) and O (42.4%) bands in $Ni(OH)_2$ in $Ni(OH)_2$, Fe (88.7%) and O (11.3%) in Fe_2O_3 and Mn (65.1%) and O (34.9) in MnO_2. Similarly, Figures S1d-f represent the MO-CNTs where in $Ni(OH)_2$-CNT (Figure S1d), Ni is at 15%, O is at 20% and C is at 64%; in Fe_2O_3 (Figure S1e) Fe is at 8%, O is at 36% and C is at 55% and; in MnO_2 (Figure S1f) Mn is at 9%, O is at 36% and C is at 54%.

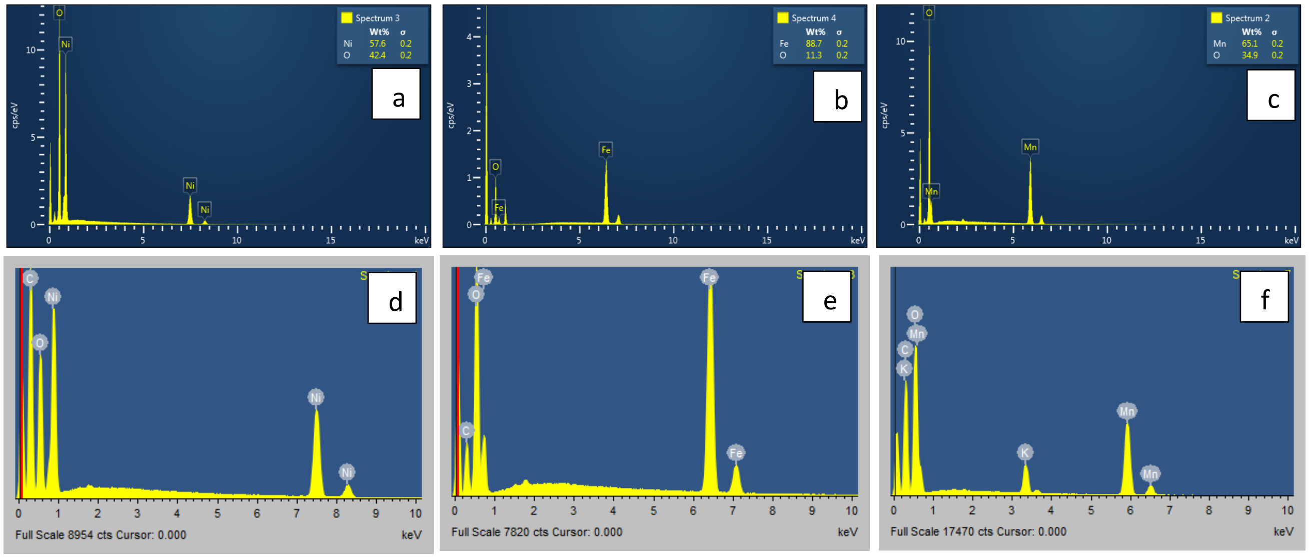

Figure S1. EDS spectra of a. $Ni(OH)_2$; b. Fe_2O_3; c. MnO_2; d. $Ni(OH)_2$-CNT; e. Fe_2O_3-CNT; f. MnO_2-CNT.
